# Impact of Acidity Profile on Nascent Polyaniline in the Modified Rapid Mixing Process—Material Electrical Conductivity and Morphological Study

**DOI:** 10.3390/ma13225108

**Published:** 2020-11-12

**Authors:** Sylwia Golba, Magdalena Popczyk, Seweryn Miga, Justyna Jurek-Suliga, Maciej Zubko, Julian Kubisztal, Katarzyna Balin

**Affiliations:** 1 Institute Materials Engineering, University of Silesia, 75 Pulku Piechoty Street 1A, 41-500 Chorzow, Poland; magdalena.popczyk@us.edu.pl (M.P.); seweryn.miga@us.edu.pl (S.M.); justyna.jurek-suliga@us.edu.pl (J.J.-S.); maciej.zubko@us.edu.pl (M.Z.); julian.kubisztal@us.edu.pl (J.K.); 2 Institute of Physics, University of Silesia, 75 Pulku Piechoty Street 1A, 41-500 Chorzow, Poland; katarzyna.balin@us.edu.pl

**Keywords:** polyaniline, polymeric conductive material, morphology, crystallinity

## Abstract

Polyaniline (PANI) was synthesized chemically with the modified rapid mixing protocol in the presence of sulfuric acid of various concentrations. A two-step synthetic procedure was utilized maintaining low-temperature conditions. Application of the modified rapid mixing protocol allowed obtaining a material with local ordering. A higher concentration of acid allowed obtaining a higher yield of the reaction. Structural characterization performed with Fourier-transform infrared (FTIR) analysis showed the vibration bands characteristic of the formation of the emeraldine salt in both products. Ultraviolet–visible light (UV–Vis) spectroscopy was used for the polaronic band and the p–p* band determination. The absorption result served to estimate the average oxidation level of PANI by comparison of the ratio of the absorbance of the polaronic band to that of the π–π* transition. The absorbance ratio index was higher for PANI synthesized in a more acidic solution, which showed a higher doping level for this polymer. For final powder products, particle size distributions were also estimated, proving that PANI (5.0 M) is characterized by a larger number of small particles; however, these particles can more easily agglomerate and form larger structures. The X-ray diffraction (XRD) patterns revealed an equilibrium between the amorphous and semicrystalline phase in the doped PANI. A higher electrical conductivity value was measured for polymer synthesized in a higher acid concentration. The time-of-flight secondary ion mass spectrometry (TOF-SIMS) analysis showed that the molecular composition of the polymers was the same; hence, the difference in properties was a result of local ordering.

## 1. Introduction

Polyaniline (PANI) belongs to the family of conducting polymers (CPs). Its properties such as controlled electroactivity, facile chemical synthesis, easy doping process, and good environmental stability place it as useful material in a range of applications including microelectronics, battery electrodes, sensors, membranes for the separation of gaseous mixtures, and tissue engineering [1,2,3,4,5]. The usage of CPs in medical areas is constantly growing as the material can be applied for monitoring vital function in living organisms, along with their stimulation [6]. There are several chemical structures of PANI chains characterized by various current conductivities, with emeraldine salt being the most conductive. The proneness of the PANI to generate charge carriers is a function of various factors influenced by the synthetic procedure [7] and by the structure of formed chains [8,9,10]. PANI is able to self-assemble into nanostructures [11], such as nanoparticles [12], nanotubes [13], nanofibers [14], nanowires [15], nanosheets [16], and networks [17]. The studies of these constructs have revealed that the electrical properties can be tuned by changing the dimensionality of the nanostructures [18,19]. PANI is synthesized via electrochemical or chemical oxidation of its monomer (aniline—ANI) in an aqueous acidic solution [20,21]. To obtain a material characterized by improved mechanical and electrical properties, one has to produce a polymer with a low number of defect sites such as chain ends and/or polymer branching along with a higher molecular weight [22]. These features can be fulfilled through usage of proper synthetic protocol. One can list the four main protocols currently used for PANI synthesis: standard protocol [6,23], rapid mixing protocol [24,25], interfacial protocol [26], and soft or hard template-based protocol [27]. In the present study, two PANI polymers were synthesized chemically using the modified rapid mixing procedure. Two different concentrations (1.0 and 5.0 M) of an inorganic acid, namely, sulfuric acid (H_2_SO_4_), were used to detect the influence of this factor on final material structure and conductivity. The novelty of the work is in its application of the modified rapid mixing protocol where the initiator (namely, ammonium peroxydisulfate) was delivered dropwise with concurrent vigorous mixing of the feed monomer solution. Such a modification of the synthesis influences the ordering of the synthesized materials. It is known that the initial acidity of the reaction solution greatly influences the acidity profile during the oxidation of the monomer [28]; an acid concentration of 1.0 M is usually used. Hence, the application of two different concentrations induces changes in the structure and morphology of formed polymers.

## 2. Materials and Methods

### 2.1. The Synthesis of Polyaniline

Synthesis was performed in two steps (main reaction and filtration) according to the modified procedure [25]. In the first step, acidic aqueous solutions of aniline (ANI (Sigma Aldrich, Poznan, Poland, ACS reagent, ≥99.5%), 0.186 g, 0.2 M) were cooled and stored at −5 °C. The temperature of the reaction mixture was steadily controlled as the oxidation of aniline is an exothermic process. Additionally, it was shown that the low temperature favored high-molecular-weight compound formation [22]. The initiator (ammonium peroxydisulfate (Sigma Aldrich, ACS reagent, ≥98.0%)—APS, 0.570 g, 0.25 M) solution was delivered dropwise to the chilled aniline solution while being vigorously mixed. This fast mixing event is a combination of the standard protocol with the rapid mixing one. The stoichiometric APS–aniline ratio was 1.25 to assure complete monomer-to-polymer conversion. After 24 h in step 2, the dark-green PANI precipitate was collected and washed twice with sulfuric acid solution ((Avantor Performance Materials Poland S.A., Gliwice, Poland, 96%), 0.2 M, 50 mL), followed by acetone (Avantor Performance Materials Poland S.A., for spectroscopy, 50 mL). Finally, polyaniline salt (emeraldine) as a powder was obtained. Excessive solvent and humidity were removed from the sample by heating at 60 °C for 24 h. The two synthesis steps were performed with various concentrations of acid (1.0 or 5.0 M), with the samples named PANI (1.0 M) and PANI (5.0 M), respectively.

### 2.2. Analytical Methods

Materials were characterized using Fourier-transform infrared (FTIR) and ultraviolet–visible light (UV–Vis) spectrophotometry. The FTIR spectra were recorded for powdered PANI at room temperature using a Shimadzu IR Prestige-21 instrument coupled with an attenuated total reflectance (ATR) head (Pike Technologies, MIRacle ATR, Madison, WI, USA) in the range of 1950–450 cm^−1^, at a resolution of 2 cm^−1^, with 100 interferograms. UV–visible spectra of PANI dispersed in *N*-methyl pyrrolidone (NMP) (c_PANI_ = 1.0 mg/mL) [29] were obtained using a Biowave II UV-visible spectrophotometer (Biochrom, Harvard Bioscience, Cambridge, UK) at room temperature with a response time of 2 s and scanning speed of 5000 nm·min^−1^. The surface morphology and chemical composition were investigated using a scanning electron microscope (JEOL JSM-6480, JEOL Ltd., Tokyo, Japan) equipped with an energy-dispersive spectroscopy (EDS) detector; a 20 kV voltage was used. Particle size analysis was performed with the use of Gwyddion 2.39 software distributed under the terms of the GNU General Public License [30]. Electrical conductivity measurements were performed using an Agilent Keysight 34401A Multimeter (Santa Rosa, CA, USA) at room temperature. The powdered dried and cooled polymers were placed between stainless-steel electrodes with a 5 mm diameter into a ceramic tube while a constant axial pressure of 3.75 MPa was applied during measurements. The four-wire electrical resistance measurement method was applied. Sample-specific electrical resistance and conductivity were calculated for the probe with a thickness of about 0.95 mm. Phase composition investigations of materials were performed using the powder X-ray diffraction (XRD) method with a Empyrean Panalytical diffractometer and CuKα (λ = 0.1542 nm) radiation equipped with a PIXcel3D detector (Malvern, UK). The data collection was over the 2θ range of 5° to 100° in 0.02° steps. Chemical composition analysis was realized with the use of time-of-flight secondary ion mass spectrometry (TOF–SIMS), a surface analytical technique that rasters a high-energy ionizing beam over a preselected area. As a result of such a sputtering process, secondary ions from a sample surface are produced. Analysis of these secondary ions allows explicitly recognizing elemental and molecular species present on the sample surface. For the above-mentioned measurements, the TOF-SIMS 5 (IONFOF GmbH, Munster, Germany) mass spectrometer was used. The spectrometer was equipped with a reflectron-type analyzer and liquid metal ion gun (Bi, 30 keV, 1 pA). Data collected from the 500 µm × 500 µm area included mass spectra measured in positive and negative polarity. Additionally, in order to neutralize the samples with the flood gun, it was necessary to adjust the parameters of charge compensation. All steady-state measurements were realized at room temperature in vacuum conditions (3 × 10^−9^ mbar). The spectra were calibrated; for positive polarity, CH_3_^+^, C_2_H_3_^+^, C_3_H_3_^+^, and C_3_H_5_^+^ ions were used, whereas, for negative polarity, C^−^, CH^−^, C_2_^−^, C_2_H^−^, C_3_^−^, and C_3_H^−^ ions were used. The SurfaceLab6 software (IONTOF GmbH Munster, Germany) was used for data analysis. The peak lists (negative and positive) were based on the results presented in [31,32,33,34].

## 3. Results

### 3.1. Polymerization and Chemical Structure Determination

#### 3.1.1. Polymerization

**Figure 1:**
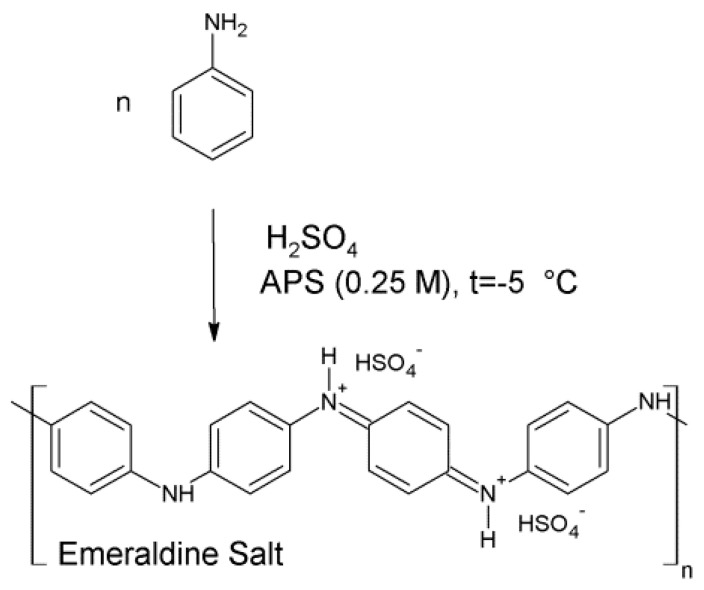
Oxidative polymerization of aniline with ammonium peroxydisulfate (APS) initiator during modified rapid mixing procedure.

Oxidative polymerization of ANI (Figure 1) occurs with a color change, going from a homogeneous, clear, and transparent reaction mixture to a dark-green suspension. At the initiation step, oxidation of the monomer occurs under the influence of APS. In the subsequent steps, radical cations undergo recombination, and fragments of any length may recombine to produce polymer chains [7].

#### 3.1.2. FTIR Analysis

**Figure 2:**
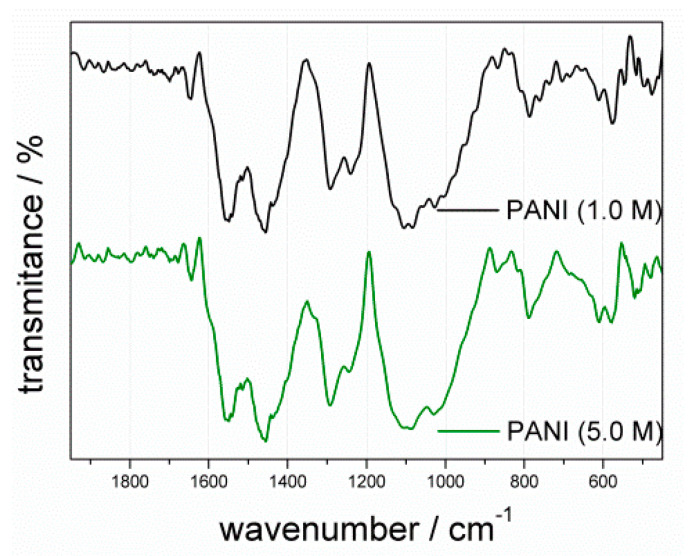
Fourier-transform infrared (FTIR) spectra of polyaniline (PANI) powders: PANI (1.0 M) (black line); PANI (5.0 M) (green line).

Powdered PANIs were subjected to chemical characterization using the FTIR technique (Figure 2), which presented the molecular structure of protonated forms of the oxidation products. The most distinctive vibration bands characteristic of the emeraldine salt of PANI were visible as the stretching modes of the benzenoid ring (B) at 1480–1500 cm^−1^ and quinoid ring (Q) at 1570–1590 cm^−1^ [20,35]. The peak at 1300 cm^−1^ corresponded to the stretching band of ν(C–N) of a secondary aromatic amine, while the peak at 1248 cm^−1^ corresponded to the stretching band of ν(C–N^+^) in the polaron lattice of PANI [36]. The shoulder band at 1140 cm^−1^ corresponded to vibration of the protonated imine (Q = NH^+^–B) or the polaron lattice of PANI (B–NH^+^–B) [37], thereby confirming the presence of the polaron form of emeraldine salt. A shoulder band at 1610 cm^−1^ was ascribed to C=C ring vibrations in the polymer chains, with symmetry broken by conformational changes induced by protonation [38]. The main chain was formed by coupling of the rings in the *para* position [7,39] as confirmed by the peak at 810 cm^−1^ corresponding to vibration of γ(C–H) in the 1,4-disubstituted aromatic ring. It was shown that not only the *para* ring position but also the *ortho* one can be activated during the propagation step [7]. This results in branched chains or intermolecular crosslinking, leading to formation of phenazine rings [40]. The *o*-coupled aniline mers with formation of phenazine-like moieties resulted in the low-intensity bands of PANI at about 1630 cm^−1^ [41,42]. Their presence was further supported by the low-intensity peak at 750 cm^−1^, reflecting vibration of γ(C–H) in the monosubstituted or 1,2-disubstituted ring, accompanied by low-intensity peaks at 700–690 cm^−1^ corresponding to out-of-plane ring bending of the monosubstituted ring which suggests irregular, branched chain formation. Additionally, the presence of the doping HSO_4_^−^ ion was ascribed to peaks at 610 and 580 cm^−1^ [42,43].

#### 3.1.3. UV–Vis Analysis

**Figure 3:**
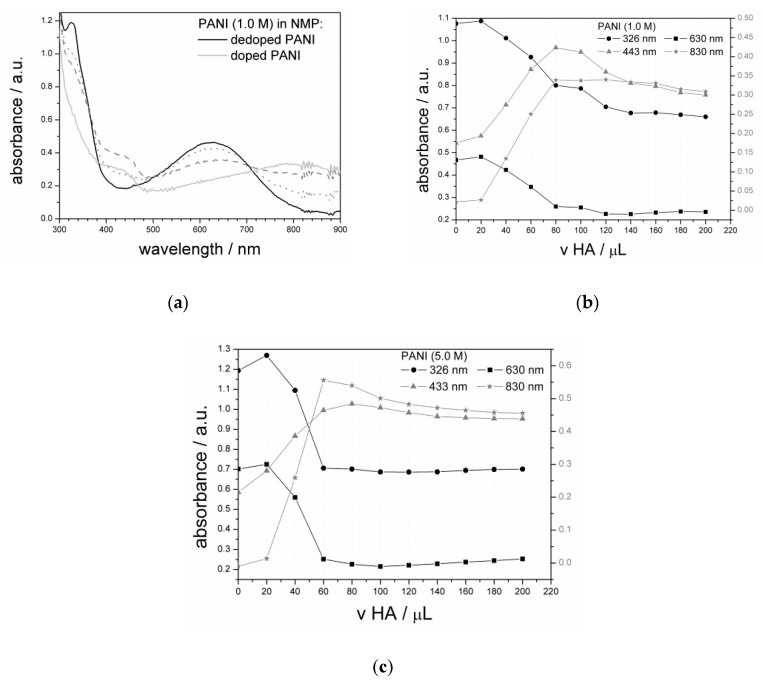
Electronic spectra of dispersion of PANI in *N*-methyl pyrrolidone (NMP): (**a**) spectra for PANI (1.0 M); (**b**,**c**) absorbance variation at chosen characteristic wavelengths during titration experiment for (**b**) PANI (1.0 M) and (**c**) PANI (5.0 M) (with H_2_SO_4_ denoted as HA).

Dispersions of PANI in NMP (1.0 mg/mL) were used to record UV–vis spectra of the dedoped and doped polymers. The spectra of dedoped PANIs revealed dual absorption bands (Figure 3a). The first (324 nm) was related to the electronic π→π* transition of electrons in benzenoid segments of pure PANI [4,44], while the second (625–630 nm) corresponded to the excitation of the imine segment in the quinoid structure of the polyaniline chain [4,45]. Subsequently, a titration experiment was performed with step dosing of a sulfuric acid solution (1.0 M). This changed the character of the spectrum with a mutual decrease in dominant band absorbance and an increase in new bands at 425–435 nm and above 730 nm (Figure 3b,c). These conditions brought modification in the structure of PANI chains with protonation of ANI units [45] as the band around 400–460 nm and the broad band spanning above 730 nm manifested localized polarons [46]. An increase in the concentration of acid elevated the doping level with an increase in absorption at the 780 nm band, associated with the biopolaronic states.

### 3.2. Morphology

**Figure 4:**
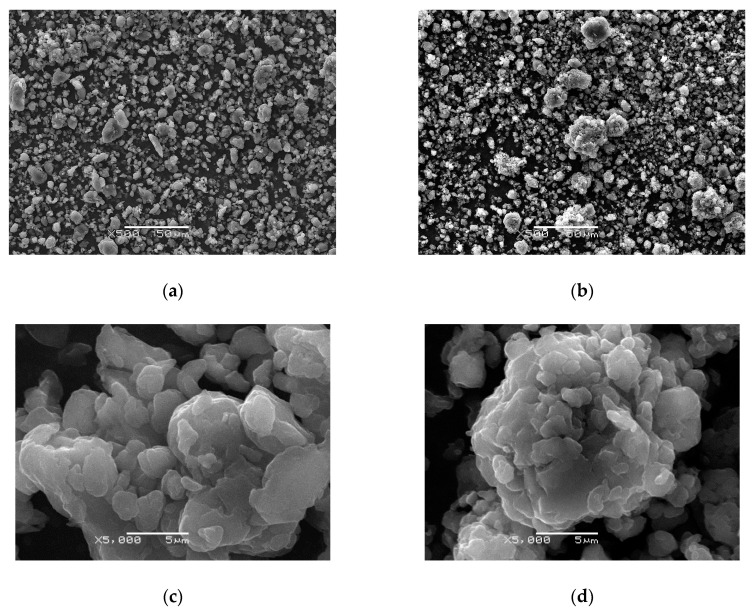
SEM images of the PANI synthesized with varying acid concentration: (**a**,**c**) PANI (1.0 M); (**b**,**d**) PANI (5.0 M). A 500× magnification is shown in (**a**,**b**), while a 5000× magnification is shown in (**c**,**d**).

The SEM observations of the morphology (Figure 4) showed that the surface of the polymers was not smooth and consisted of a heterogeneous and granular structure formed by the aggregation of small flake-like structures. Typically, one can find that PANIs are composed of “cauliflower” structures [46,47,48], which refer mainly to their amorphous morphology. Here, clusters were also found; however, the components were flattened in both cases. The sulfuric acid concentration was found to affect PANI morphology. For PANI (1.0 M), the particles were smaller with a more flake-like two-dimensional structure surface (Figure 4a) (similar to that seen in low-molarity sulfuric acid [47]), while, in the case of PANI (5.0 M) (Figure 4b), larger particles were observed with a more globular structure that showed better cohesion and higher aggregation.

**Figure 5:**
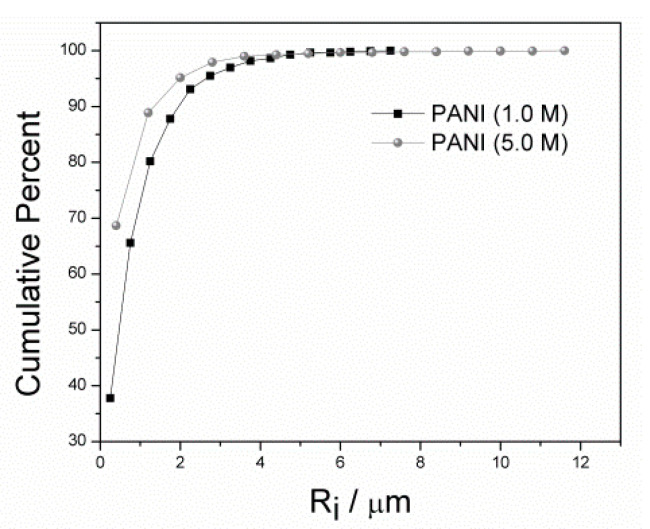
Cumulative percentage of particles from PANI (1.0 M) and PANI (5.0 M) samples calculated on the basis of the images presented in Figure 4a,b; R_i_ is the maximum radius of a circle inscribed into a particle.

As the final product was obtained in the form of a powder, particle size distributions were estimated using the R_i_ parameter, i.e., the maximum radius of a circle inscribed into a particle; the results are presented in Figure 4a,b The cumulative percentage of particles obtained is shown in Figure 5. It was found that, for PANI (5.0 M), the number of particles with R_i_ below 1 µm was ca. 10% higher in comparison with PANI (1.0 M). Moreover, the R_i_ parameter did not exceed ca. 12 µm and 7 µm for PANI (5.0 M) and PANI (1.0 M), respectively. Thus, it can be stated that PANI (5.0 M) was characterized by a larger number of small particles; however, these particles could more easily agglomerate and form larger structures.

The chemical composition of both polymers was studied using EDS analysis (Table 1), which confirmed the presence of HSO_4_^−^ doping ions (the measured content is approximate). The results showed the presence of 6.4 wt.% sulfur for PANI (1.0 M) and 4.2 wt.% sulfur for PANI (5.0 M) indicating the presence of remaining oxidation agent in the sample.

### 3.3. Crystallinity

**Figure 6:**
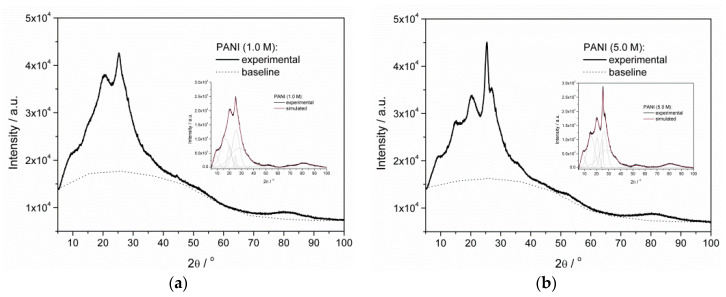
X-ray diffraction (XRD) pattern of doped (**a**) PANI (1.0 M) and (**b**) PANI (5.0 M) powders and spectral deconvolution (inserts).

The XRD patterns (Figure 6) and the corresponding peak distribution revealed a central broad band for both samples, confirming their prevailing amorphous nature. The diffraction peaks observed at 2θ = 15.00°, 25.30°, and 20.57° could be prescribed to the (011), (020), and (200) crystal planes of PANI [49,50,51], implying the presence of a material with a local, short distance arrangement in the structure. According to the literature, 2θ = 28.72° and 22.74° correspond to the periodicity parallel and perpendicular to the polymer chains of PANI, respectively [46,52], while the peak at 2θ = 25.42° is a characteristic peak of the emeraldine salt [53].

Broad peaks proved the local structural ordering of a material characteristic of polymers with a low degree of crystallinity, resembling a fringed micelle model [54]. The order of polymer crystallinity can be estimated with the ratio of half-width to height (HW/H) of an X-ray diffraction peak [55]. The analysis of XRD results (Table 2) indicated the contribution of the amorphous part of the polymers and revealed that the width-to-height ratio for diffraction peaks characteristic of an ordered structure was lower for PANI (5.0 M). This confirmed that this material had a less amorphous nature in comparison to PANI (1.0 M).

### 3.4. Electrical Conductivity

PANI is a heterogeneous system where the ordered regions are dispersed among disordered ones. The ordered domains allow for conduction through electron delocalization or hopping of charge carriers [56]. The room temperature resistance of powdered samples allowed the specific electrical resistance (ρ) and electrical conductivity (σ) to be calculated, with results of ρPANI (1.0 M) = 26.78 Ω∙m and ρPANI (5.0 M) = 23.38 Ω∙m, and σPANI (1.0 M) = 0.037 S∙m^−1^ and σPANI (5.0 M) = 0.043 S∙m^−1^, respectively. It was apparent that the conductivity of PANI (5.0 M) was slightly higher than that of PANI (1.0 M).

### 3.5. TOF-SIMS Mass Spectra of PANIs

**Figure 7:**
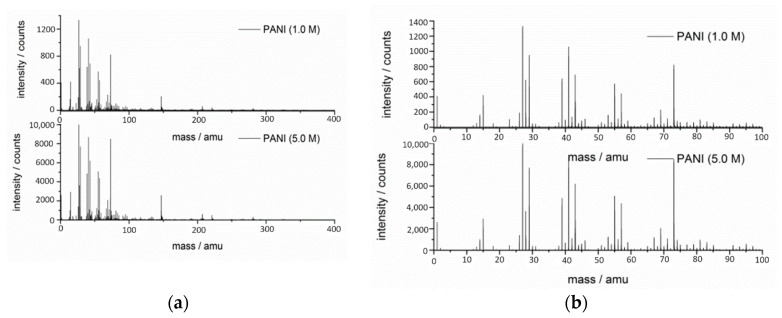
Time-of-flight secondary ion mass spectrometry (TOF-SIMS) mass spectra of doped PANI (1.0 M) and PANI (5.0 M) in positive polarity of the mass analyzer: (**a**) 0–400 amu range; (**b**) 0–100 amu range.

**Figure 8:**
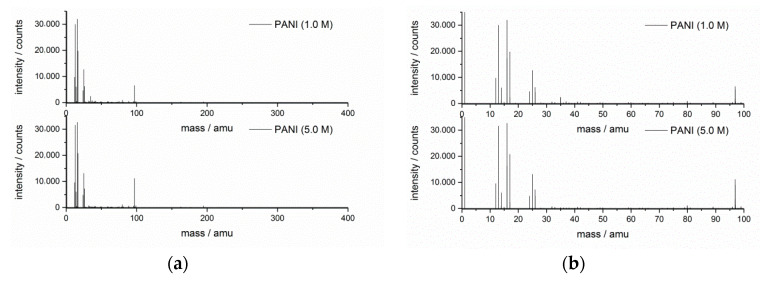
TOF-SIMS mass spectra of doped PANI (1.0 M) and PANI (5.0 M) in negative polarity of the mass analyzer: (**a**) 0–400 amu range; (**b**) 0–100 amu range.

The TOF-SIMS surface analytical technique was used to investigate the surface composition of the polymers. Secondary ions were detected in both positive and negative modes; for both polarities, a full spectrum in a range of 1–1200 amu was acquired. Figure 7 shows the mass spectra of PANIs presenting the positively charged ions with a wide 0–400 amu and narrow 0–100 amu mass range, while Figure 8 shows the negatively charged ions in the same mass ranges. The mass spectra of both PANIs showed similar characteristic peaks and similar intensities, as the carbon skeleton for both polymers was the same. The positive ion spectra revealed fragments of up to 300 amu for both polymers with the spectrum of PANI (5.0 M) being more intense, while, for negative ion spectra, fragments of up to 200 amu were revealed. The positively charged ions within the range of 0–100 amu (Figure 7a,b) revealed several characteristic peaks corresponding to aliphatic hydrocarbon fragments of the PANI polymer chain. Hence, peaks representing fragments based on patterns such as C_n_H_2n−3_, C_n_H_2n-1_, and C_n_H_2n+1_ were found. Table 3 presents the assignment of the selected peaks of PANI detected in positive and negative polarity. Along with a change in analyzer polarity to negative, the most intense ion peaks detected at *m*/*z* = 26, 42, and 50 amu (Figure 8a,b) were correlated to the CN^−^, CNO^−^, and C_3_N^−^ fragments, characteristic of the PANI fragmentation path [34]. The intensive HSO_4_^−^ (96.96 amu) peak presumably came from the residual doping ion.

## 4. Discussion

According to the amount of the final product, efficiency of the polymerization reaction was calculated according to Equation (1).

(1) $$E=\frac{m_{\mathrm{polymer}}}{m_{\mathrm{monomer}}}\times 100\%.$$

The calculated values were 57.9% for PANI (1.0 M) and 63.0% for PANI (5.0 M), indicating that a higher concentration of acid slightly improved the efficiency of the reaction. As sulfuric acid is diprotic, its dissociation in water is a two-stage process with the formation of several products, namely, a hydronium ion, hydrogen sulfate ion (HSO_4_^−^), and sulfate ion (SO_4_^2−^). Hence, there are at least two anionic species able to compensate for the cationic charge in protonated oligomeric or polymeric chains. In the more concentrated acid (5.0 M) solution, there were more H_3_O^+^ ions coming from the dissociation process. The additional number of dissociated ions enhanced the nucleation stage via the formation of a larger amount of anilinium cations [42], leading to a more effective protonation of previously formed chains. Such behavior led to higher coupling efficiency and, hence, to higher yield of the reaction. This also potentially increased the rate of a further recombination step, leading to a decrease in the mass of the formed macromolecules. Additionally, one must bear in mind that, in accordance with the reaction shown in Figure 1, there were two sources of SO_4_^2−^ doping ions. One was from the added H_2_SO_4_ acid, while the second was from peroxysulfate ion (S_2_O_8_^2−^) dissociation into the sulfate radical anion and hydroperoxosulfate ion before transformation into a hydrosulfate ion [57]. Comparison of the intensities of IR bands specific to vibrations of Q and B units (I_B_/I_Q_) allowed estimating the molar ratio of these structures with the preliminary assumption of the molar absorption coefficient being the same in both cases [35,48,58]. For PANI (1.0 M), the I_B_/I_Q_ ratio was 1.20, while that for PANI (5.0 M) was 1.12, indicating that the polymer obtained in more acidic conditions contained more quinoid moieties.

The absorption result served to estimate the average oxidation level of PANI through a comparison of the ratio of absorbance of the polaronic band (A_830–850_) to that of the π–π* transition (A_326_) [59]. For the studied samples, the absorbance ratio index calculated as A_830_/A_326_ was 0.489 for PANI (1.0 M) and 0.659 for PANI (5.0 M) (Figure 3), showing a higher doping level for PANI (5.0 M). This conclusion was also supported by the higher intensity of the absorbance band, corresponding to charge carrier moieties for the PANI (5.0 M) sample.

The higher aggregation state of the synthesized material (shown in SEM images) was due to the physical and chemical properties of the solution and rate of polymerization. As shown by Sapurina [7], as the initial pH of the reaction medium decreases, the temperature increases markedly. This is related to the exothermic nature of the polymerization reaction, especially at the stage of initiation (oxidation reaction and further proton liberation). For a highly acidic medium such as the 5.0 M solution of H_2_SO_4_, the initial oxidation rate was high with a shorter oxidation induction period observed at the beginning of the process. In the studied cases, the temperature of the reaction medium was maintained at −5 °C, and the reaction started earlier for the 5.0 M solution. The higher concentration of acid evoked a more profound increase in temperature leading to a shorter induction period and a presumably higher rate of oxidation. As a result, more chains were formed and aggregated in a more dense, compact structure visible in the SEM image (Figure 4d). Moreover, in the case of 1.0 M, synthesis of a more loosely packed structure was presented (Figure 4c). A high acid concentration seems to favor nucleation site formation, which further enhances three-dimensional (3D) nanoparticle agglomeration at the initial and secondary growth stages [60]. Hence, a more compact structure was found in this case. It was also shown that the presence of dopant ions imparted both hydrophilicity and solubility to PANI [61]. The SEM images of both PANIs (Figure 4a–d) demonstrate that the grains were packed but still distinguishable, pointing to their hydrophobic nature.

A local organization of polymer chains was produced in response to charge carrier formation. The production of a charge on the chain led to electrostatic repulsion between the neighboring chains, leading to straightening of the polymer chains. The partial loss of flexibility provided adequate conditions for local arrangement in close proximity. The average domain length or extent of order (L) of PANI chains could be estimated using Scherrer’s formula (Equation (2)) [62,63].

(2) $$L=\frac{K\lambda }{\beta \cos\theta }$$

where K is the proportionality constant (equal to 0.9 for unknown structures), β is the full width at half maximum (HW) expressed in radians, and λ (= 1.5406 Å) is the wavelength of X-rays used.

For deeper characterization of the structural composition of the samples, the interplanar spacing (d) and interchain separation length (R) of PANI samples could also be calculated [26,48,63]. The value of interplanar spacing (d) was defined according to the Bragg relation (Equation (3)), while the Klug and Alexander equation was used for calculation of the interchain separation length (Equation (4)),

(3) $$d=\frac{\lambda }{2\sin\theta }$$

(4) $$R=\frac{5\lambda }{8\sin\theta }$$

The values of the above-mentioned parameters for PANI samples were calculated at the most intensive 2θ angle, resulting in 25.514 for PANI (1.0 M) and 26.008 for PANI (5.0 M). The average domain length (L) for PANI (1.0 M) was equal to 1.208 nm, while that for PANI (5.0 M) was equal to 1.410 nm. The results showed that the average domain length increased for PANI (5.0 M), supporting the idea of a longer conjugation length obtained for this sample. Moreover, both parameters (i.e., interplanar spacing (d) and interchain separation length (R)) were smaller for PANI (5.0 M) with the corresponding values equal to d_PANI_ (1.0M) = 3.488 Å, R_PANI_ (1.0 M) = 4.360 Å and d_PANI_ (5.0 M) = 3.413 Å, R_PANI_ (1.0 M) = 4.266 Å. These values clearly show that the chains of PANI (5.0 M) were less loosely packed, enabling easier charge carrier hopping. The calculated values are comparable to those available in the literature [63], leading to a conclusion of low crystallinity of the obtained samples.

The obtained conductivity values of PANIs were moderate in comparison to the literature value of conductivity for PANI-doped materials (10 S∙cm^−1^) [50,64]. The electrical conductivity mainly depends on the type, number, and mobility of the charge carriers. In the PANI powder, charge carriers must be transferred through the polaron structure generated upon protonation of the nitrogen group in the ordered regions. In the bulk structure of the material, ordered regions are embedded in an amorphous nonconducting region of entangled chains. Hence, in the studied cases, the overall conductivity of PANI depended on the connection between conductive regions. In the synthesis performed in 5.0 M acid, these regions were less disordered (as shown by XRD analysis); hence, the electrical conductivity was higher. It seems that, although the complexity of the TOF-SIMS spectra was higher for positive polarity, it is the negative polarity that allowed probing more nitrogen-based specific molecular fragments bringing some structural information. According to [65], in the course of aniline polymerization, several side products, as well as the product of the subsequent condensation reaction, can be formed. On the basis of the TOF-SIMS result, one may conclude that the molecular compositions of the synthesized samples were very similar, and the variation in their physical properties was the outcome of the local ordering as opposed to the presence of such side or condensation products.

## 5. Conclusions

Powdered polyaniline (PANI) was chemically synthesized with two different acid concentrations. The two-step synthetic procedure allowed producing a powdered material insoluble in the reaction environment. The concentration of acid influenced both the efficiency of the reaction and the conductivity of final polymers. Spectral analysis showed that the conductive form of the polymer was synthesized, as signals of both benzenoid (B) and quinoid (Q) rings accompanied by peaks corresponding to characteristic features of the polaron lattice were detected. Furthermore, signals of the doping ion were found. The electronic spectra confirmed the presence of characteristic bands of dedoped PANI, with the titration experiment revealing characteristic bands of doped PANI. The average oxidation level of PANI (5.0 M) was higher according to the absorbance ratio index.

The morphology of the PANI powder was influenced by the acid concentration, with smaller particles presenting a flake-like surface formed in the less concentrated solution and larger particles with a more globular structure formed in the more concentrated solution. The analysis of XRD peak distribution revealed a less disordered structure with partial arrangement of the macromolecules of PANI (5.0 M). These results indicate the vital role of acidic conditions during PANI synthesis. A higher conductivity was also calculated for PANI (5.0 M) in comparison to PANI (1.0 M). This indicates that a more ordered structure favors hopping of the charge carrier in ordered domains. TOF-SIMS analysis showed that the molecular composition of the polymers was the same; hence, the difference in properties was the result of more local ordering as opposed to the presence of condensation products.

## Figures and Tables

**Table 1:** Elementary composition of PANIs based on energy-dispersive spectroscopy (EDS) analysis.

PANI	Elements			
	C	N	O	S
	at.%	at.%	at.%	at.%
1.0 M	66.3	20.0	7.2	6.4
5.0 M	62.7	23.6	9.5	4.2

**Table 2:** XRD data analysis of PANI (1.0 M) and PANI (5.0 M).

Sample	2θ (°)	HW ^1^ (°)	H ^2^ Intensity	HW/H Ratio
PANI (1.0 M)	9.136	4.216	4258.17	9.90 × 10^−4^
	16.725	9.057	10,222.62	8.86 × 10^−4^
	20.328	3.607	7563.24	4.77 × 10^−4^
	25.244	1.341	4374.34	3.07 × 10^−4^
	25.514	6.738	13,225.05	5.09 × 10^−4^
	28.127	16.458	6789.06	2.42 × 10^−3^
PANI (5.0 M)	9.034	3.831	4787.73	8.00 × 10^−4^
	15.424	6.827	11,261.78	6.06 × 10^−4^
	20.491	3.553	10,899.39	3.26 × 10^−4^
	25.273	0.974	10,620.53	9.17 × 10^−5^
	26.088	5.783	12,943.19	4.47 × 10^−4^
	28.269	16.317	6260.73	2.61 × 10^−3^

^1^ HW—peak half-width; ^2^ H—peak height.

**Table 3:** Selected peaks and their assignment observed in positive- and negative-ion time-of-flight secondary ion mass spectrometry (TOF-SIMS) spectra of investigated samples.

Positive Ion Mass Spectrum, *m*/*z*	Probable Ion Fragment	Positive Ion Mass Spectrum, *m*/*z*	Probable Fragment	Negative Ion Mass Spectrum, *m*/*z*	Probable Ion Fragment
15.0227	CH_3_^+^	57.0782	C_4_H_9_^+^	26.0048	CN^−^
27.0228	C_2_H_3_^+^	67.0584	C_5_H_7_^+^	41.9994	CNO^−^
29.0412	C_2_H_5_^+^	69.0781	C_5_H_9_^+^	50.0068	C_3_N^−^
39.0214	C_3_H_3_^+^	73.0583	C_3_H_7_NO^+^		
41.0404	C_3_H_5_^+^	81.0774	C_6_H_9_^+^		
43.0587	C_3_H_7_^+^	83.0952	C_6_H_11_^+^		
55.0588	C_4_H_7_^+^	95.0957	C_7_H_11_^+^		
